# Regulon analysis identifies protective FXR and CREB5 in proximal tubules in early diabetic kidney disease

**DOI:** 10.1186/s12882-023-03239-6

**Published:** 2023-06-19

**Authors:** Wanting Shi, Weibo Le, Qiaoli Tang, Shaolin Shi, Jingsong Shi

**Affiliations:** 1 National Clinical Research Center for Kidney Disease, Affiliated Jinling Hospital, Medical School, Nanjing University, Nanjing, China; 2 Department of Nephrology, the First Affiliated Hospital of University of Science and Technology of China, Hefei, China

**Keywords:** Diabetic kidney disease, Single nucleus RNA-seq, Gene regulatory network, Proximal tubule epithelial cells, FXR, CREB5

## Abstract

Diabetic kidney disease (DKD) is the most common complication of diabetes mellitus and a leading cause of kidney failure worldwide. Despite its prevalence, the mechanisms underlying early kidney damage in DKD remain poorly understood. In this study, we used single nucleus RNA-seq to construct gene regulatory networks (GRNs) in the kidney cortex of patients with early DKD. By comparing these networks with those of healthy controls, we identify cell type-specific changes in genetic regulation associated with diabetic status. The regulon activities of FXR (NR1H4) and CREB5 were found to be upregulated in kidney proximal convoluted tubule epithelial cells (PCTs), which were validated using immunofluorescence staining in kidney biopsies from DKD patients. In vitro experiments using cultured HK2 cells showed that FXR and CREB5 protected cells from apoptosis and epithelial–mesenchymal transition. Our findings suggest that FXR and CREB5 may be promising targets for early intervention in patients with DKD.

**Supplementary Information:**

The online version contains supplementary material available at 10.1186/s12882-023-03239-6.

## Introduction

Diabetic kidney disease (DKD) develops in approximately 40% of diabetic patients and has become the most common cause of chronic kidney disease worldwide [1]. Early intervention is critical in preventing progression toward kidney failure. DKD manifests early pathological changes in both glomerulus and tubulointerstitium, but the regulatory mechanism remains poorly understood. Single cell/nucleus transcriptomics of the kidney has emerged in recent years, which has provided an opportunity to investigate the cell type specific mechanisms of kidney disease.

We used the single nucleus RNA-seq data and Single-Cell rEgulatory Network Inference and Clustering (SCENIC) method to construct a gene regulatory network in the kidneys of patients with early DKD. SCENIC is a powerful tool for simultaneous reconstruction of gene regulatory networks and identifying cell states [2]. It incorporates transcription factor information into gene coexpression modules and quantifies the activity of subnetworks in each cell, allowing for the comparison of cell-specific networks among different cell types and cell states.

We identified prominent activated regulons in proximal tubular epithelial cells (PCTs), collecting duct principal cells (CD-PCs) and glomerular endothelial cells. Since PCTs play a crucial role in the pathogenesis and progression of DKD [3, 4], we focused on farnesoid X receptor (FXR) and cAMP response element-binding protein 5 (CREB5), which are specifically activated in diabetic PCTs. We validated their expression in PCTs of early DKD patients and determined their role in apoptosis and epithelial–mesenchymal transition (EMT) in the human PCT cell line HK2.

## Materials and methods

### Single nucleus RNA-seq data preprocessing

We used the single nucleus RNA-seq dataset GSE131882 [5] from the NCBI GEO database. This dataset includes 3 early human diabetic kidney samples and 3 controls. Quality control (QC) filters were applied using the following parameters: (1) genes detected in < 3 cells were excluded; (2) cells with < 500 or > 10,000 genes were excluded; (3) cells with > 15% mitochondrial RNA reads were excluded; (4) mitochondrial RNAs were excluded; and (5) 243 doublet artifacts were removed with scDblFinder [6]. After the QC filters, a total of 21,785 cells and 36,480 genes were retained. Seurat v4.0.1 was used for single nucleus data analysis [7]: The raw read counts were normalized per cell using the "NormalizeData" function by dividing the total number of reads in that cell, then multiplying by a scale factor of 10,000 and taking natural log transformed values. We selected 2000 highly variable genes based on the average expression and dispersion per gene using the "FindVariableFeatures" function with parameters. Then, we selected features that are repeatedly variable across datasets, identified anchors using the "FindIntegrationAnchors" function, and used these anchors to integrate the 6 samples together with the "IntegrateData" function.

### Unsupervised clustering and cell type identification

After data scaling, principal component analysis was performed on the highly variable genes using the RunPCA function. The top 30 principal components were chosen for cell clustering, and the Uniform Manifold Approximation and Projection (UMAP) plot is shown. Cells were clustered using the "FindClusters" function (Leiden algorithm) with resolution = 0.5. Each cluster was screened for marker genes by differential expression analysis (DEA) between cells inside and outside the cluster using the "FindAllMarkers" function with parameters min.pct = 0.25 (genes expressed in at least 25% of cells either inside or outside of a cluster) and test.use = “wilcox” (Wilcoxon rank sum test), and only positive makers were retained. Compared with canonic cell type markers in published papers [7, 8], the 22 cell clusters were identified to 15 cell types. Differentially expressed (DE) genes in each type of cell between the diabetic and control patients were chosen satisfying the following criteria: (1) adjusted *p*-value < 0.05; (2) log fold change > 0.25; and (3) the percentage of cells that expressed the gene in either of the two populations > 25%.

### Gene regulatory network construction

SCENIC analysis (pyscenic version 0.10.3) was performed on all single cells to build a transcriptional regulatory network [9]. First, coexpression modules are inferred using a regression per-target approach (GRNBoost2). Next, the indirect targets are pruned from these modules using cis-regulatory motif discovery (cisTarget). Finally, the activity of these regulons is quantified via an enrichment score for their target genes (AUCell). The differentially activated regulations were calculated using the Wilcoxon rank sum test and filtered using the following parameters: (1) adjusted *p* value < 0.01; (2) difference in activity score > 0.02. Only regulons specifically activated in at least one cell type and significantly upregulated or downregulated in early diabetic kidneys were involved in further analysis.

### Patient enrollment and immunofluorescence staining

All DKD cases were diagnosed based on kidney biopsies performed at the National Clinical Research Center of Kidney Diseases, Medical School of Nanjing University. Kidney biopsies from 5 DKD patients and 6 MCD patients, were obtained from this center’s kidney biorepository. The frozen sections were prepared by cutting the tissue into 5 μm sections with a cryostat (Leica, CM1950) and kept at –80 °C until use. For staining, the sections were washed 3 times in PBS at room temperature followed by blocking buffer (5% BSA) for 30 min at room temperature. Primary antibodies for FXR (Proteintech, 25,055–1-AP) and CREB5(Bioss, bs-14053R) were diluted in PBS and applied to the section followed by incubation at 4 °C overnight. The next day, the slides were washed 3 times for 10 min with TBST, and then a secondary antibody solution was applied and incubated for 1 h at room temperature, washed 3 times for 10 min with TBST. Then slides were stained with DAPI for 10 min at room temperature and washed 3 times for 10 min with TBST and briefly washed with water before mounting (Dako, S3023). The staining images were taken using identical imaging parameters with a confocal laser microscope (Zeiss). Staining intensities and the distribution patterns were compared under the same conditions.

### In vitro study

HK2 cells, a cell line derived from human proximal tubular cells, were purchased from the American Type Culture Collection (ATCC®CRL-2190™). The cells were cultured in DMEM containing 10% fetal bovine serum and 1% penicillin–streptomycin (100 U/ml of each) (Biosharp, BL505A) and grown at 37 °C. After subculturing and growth to 50–60% confluence, siRNA targeting FXR or CREB5 was transferred into HK2 cells by Lipofectamine® RNAiMAX reagent according to the manufacturer’s instructions. The medium was changed after 12 h, and the cells were treated with AGE (Bioss, bs-1158P) or palmitic acids (Kunchuang Biotech, KT002). All groups of cells were harvested 24 or 36 h after transfection or at the indicated time. The siRNA sequences of FXR and CREB5 are listed in Supplemental Table 1. For western blotting, HK2 cells in 6-well plates were washed with cold PBS and then lysed with 150 μl of RIPA buffer per well (BOSTER, AR0102-100) containing proteinase inhibitor cocktail (Roche, 04693116001) and phosphatase inhibitors (Roche, 04906837001). The antibodies used for western blotting included FXR (proteintech, 25055-1-AP), CREB5 (Bioss, bs-14053R), cleaved caspase-3 (Cell signaling, 05/2016), cleaved PARP1 (Proteintech, 66520-1-Ig), fibronectin (Proteintech, 66042-1-Ig), vimentin (Proteintech,10366-1-AP), E-cadherin (Proteintech, 20874-1-AP), GAPDH (Proteintech, 60004-1-Ig), and beta-tubulin (Bioworld, AP0064).

### Statistical analysis

The obtained data from each experiment were expressed as the mean ± SEM. Statistical comparisons between the groups were performed using Student’s t-test, and statistical significance was set at *p* < 0.05.

## Results

### Cell type identification

**Fig. 1:**
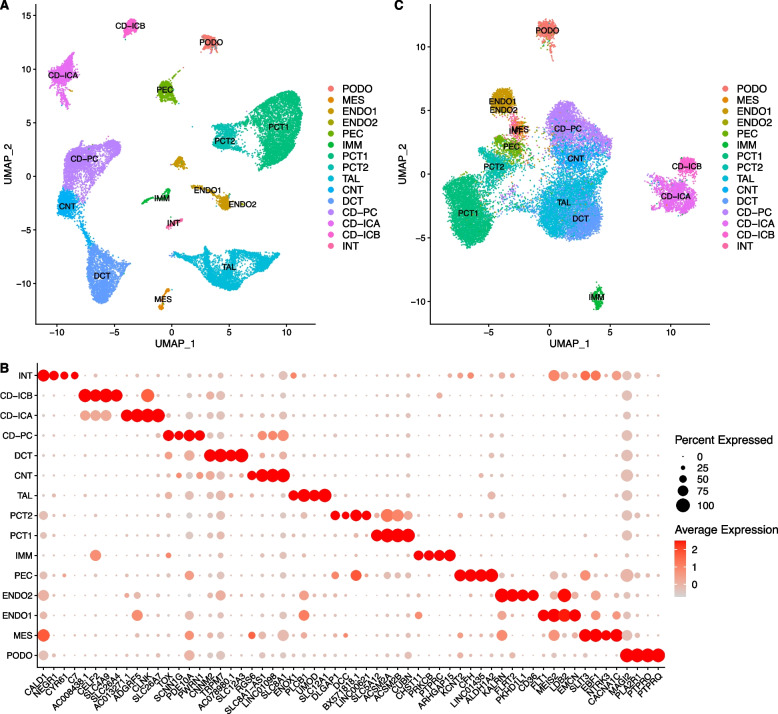
Highly variable genes and SCENIC UMAP plots with cell type classifications. **A** UMAP visualization of cells using the same principal components as input to the clustering analysis; **B** Dot plot of selected marker genes within each cell type. The size of the dot encodes the percentage of cells within a cell type, while the color encodes the average expression level across all cells within a cell type; **C** UMAP visualization of cells using the SCENIC regulon activity matrix. PODO, podocytes; MES, mesangial cells; ENDO1, glomerular endothelial cells; ENDO2, endothelia cells type 2; PEC, parietal epithelial cells; IMM, immune cells; PCT1, proximal tubule epithelial cells type 1; PCT2, proximal tubule epithelial cells type 2; TAL, thick ascending limb cells; CNT, connecting tubule cells; DCT, distal convoluted tubule cells; CD-PC, collecting duct principal cells; CD-ICA, collecting duct intercalated cells type A; CD-ICB, collecting duct intercalated cells type B; INT, interstitial cells

Single nucleus transcriptome data GSE131882 from human kidney cortex samples (including 3 early DKD samples and 3 healthy control samples) were downloaded from the NCBI GEO database. After data cleaning, quality control, exclusion of mitochondrial genes, and doublet removal, a total of 21,785 cells and 36,480 genes were retained (Materials and Methods). A total of 21,785 cells were divided into 22 clusters using the Leiden algorithm [10]. By canonical cell type markers, the 22 cell clusters were identified as 15 cell types (Table 1, Fig. 1A). By sorting the fold changes of genes expressed inside and outside the cell types in descending order, the top four differentially expressed (DE) genes were selected as marker genes for each cell type (Fig. 1B).

**Table 1:** Cell types and their numbers

Index	Abbr	Cell type	Cell number
1	PODO	Podocytes	630
2	MES	Mesangial cells	238
3	ENDO1	Glomerular endothelial cells	1033
4	ENDO2	Endothelial cells type 2	31
5	PEC	Parietal epithelial cells	718
6	IMM	Immune cells	336
7	PCT1	Proximal convoluted tubule epithelial cells type 1	4404
8	PCT2	Proximal convoluted tubule epithelial cells type 2	1039
9	TAL	Thick ascending limb cells	3687
10	CNT	Connecting tubule cells	1289
11	DCT	Distal convoluted tubule cells	2688
12	CD-PC	Collecting duct principal cells	568
13	CD-ICA	Collecting duct intercalated cells type A	1667
14	CD-ICB	Collecting duct intercalated cells type B	568
15	INT	Interstitial cells	250

### Construction of gene regulatory networks

**Fig. 2:**
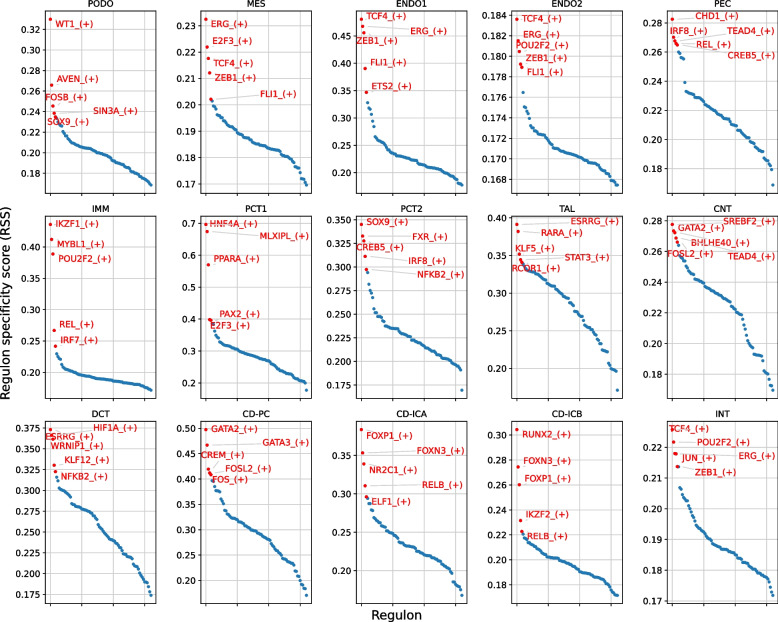
RSS panel plot with all cell types. The top 5 regulons from each cell type are marked in red and annotated. PODO, podocytes; MES, mesangial cells; ENDO1, glomerular endothelial cells; ENDO2, endothelia cells type 2; PEC, parietal epithelial cells; IMM, immune cells; PCT1, proximal tubule epithelial cells type 1; PCT2, proximal tubule epithelial cells type 2; TAL, thick ascending limb cells; CNT, connecting tubule cells; DCT, distal convoluted tubule cells; CD-PC, collecting duct principal cells; CD-ICA, collecting duct intercalated cells type A; CD-ICB, collecting duct intercalated cells type B; INT, interstitial cells

To construct the gene regulatory networks for all major kidney cell types, we applied the SCENIC pipeline. The main outcomes include a regulon activity matrix, in which the columns represent the cells, and the rows represent the regulons (each representing a TF along with a set of coexpressed and motif significantly enriched target genes). The UMAP plot of the regulon activity matrix (Fig. 1C) shows that the different types of kidney cells were well separated, and the distribution of cell types was in accordance with the anatomic location. To identify critical regulators for each cell type, we evaluated each regulon’s activities associated with all 15 cell types, which were defined as regulon specificity scores (RSSs) [11, 12]. The top five regulators for the maintenance of cell identity are shown in Fig. 2.

### Disease related regulons

**Fig. 3:**
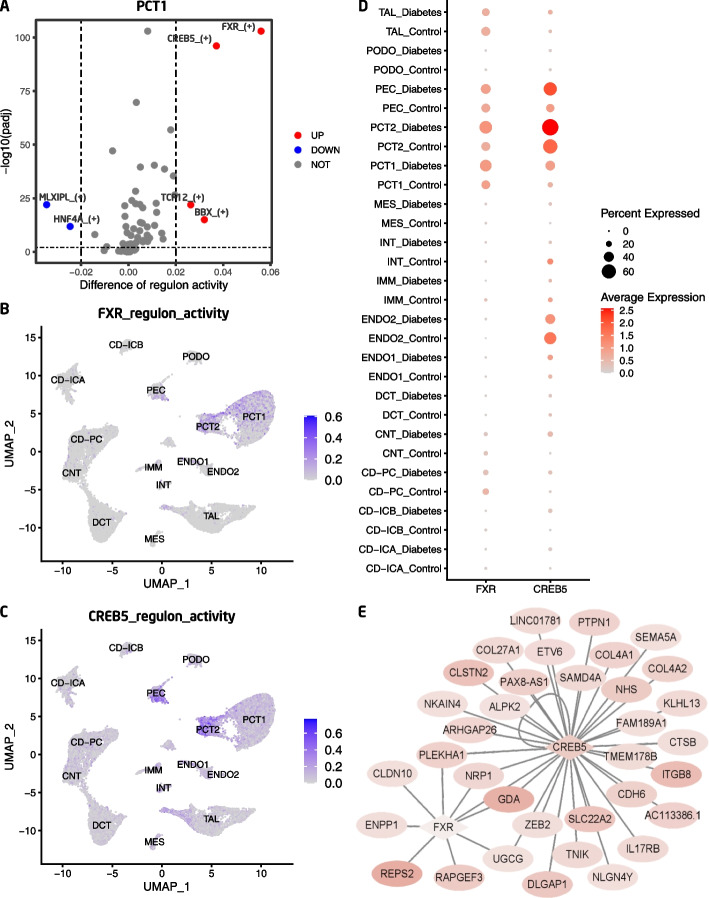
**A** The regulon activity changes in kidney proximal convoluted tubule epithelial cell type 1 in diabetic patients; PCT1, proximal convoluted tubule epithelial cell type 1; **B** The regulon activities of FXR (NR1H4) across kidney cell types; **C** The regulon activities of CREB5 across kidney cell types; **D** mRNA expression of FXR and CREB5 across kidney cell types in diabetic and control conditions; **E** Gene regulatory network of the TFs FXR (NR1H4) and CREB5 and their target genes. The pink color of a shade indicates that the gene was upregulated in diabetic PCT cells, and the intensity of shade denotes the relative change across genes

We further compared the difference in regulon activity values in each type of cell between the diabetic and control conditions. This is important because the differentially activated TFs and their target genes might play important roles in DKD. The regulon activities of FXR and CREB5 were increased in the proximal tubule epithelial cells (PCT1 and PCT2) of DKD patients compared with the control group (Fig. 3A-C, Supplemental Fig. 1). Consistently, the mRNA levels of FXR and CREB5 were relatively high in the PCT cells and upregulated in the diabetic group (Fig. 3D). The gene regulatory network of FXR and CREB5 is shown in Fig. 3E. The regulon activities of CREM and FOSL2 were upregulated in the diabetic CD-PC cells compared with the control group (Supplemental Fig. 2A-C), and the mRNA expressions of CREM and FOSL2 were also upregulated accordingly (Supplemental Fig. 2D). The gene regulatory network of CREM and FOSL2 and their target genes are shown in Supplemental Fig. 2E. The regulon activity and mRNA expression of TCF4 were specifically upregulated in the diabetic endothelial cells compared with the control group (Supplemental Fig. 3A-B), and the mRNA levels of TCF4 were relatively high in the endothelial cells and upregulated in the diabetic group (Supplemental Fig. 3C). The gene regulatory network of TCF4 is shown in Supplemental Fig. 3D.

### Immunostaining confirmed increased proteins of FXR and CREB5 in PCT cells of diabetic patients

**Fig. 4:**
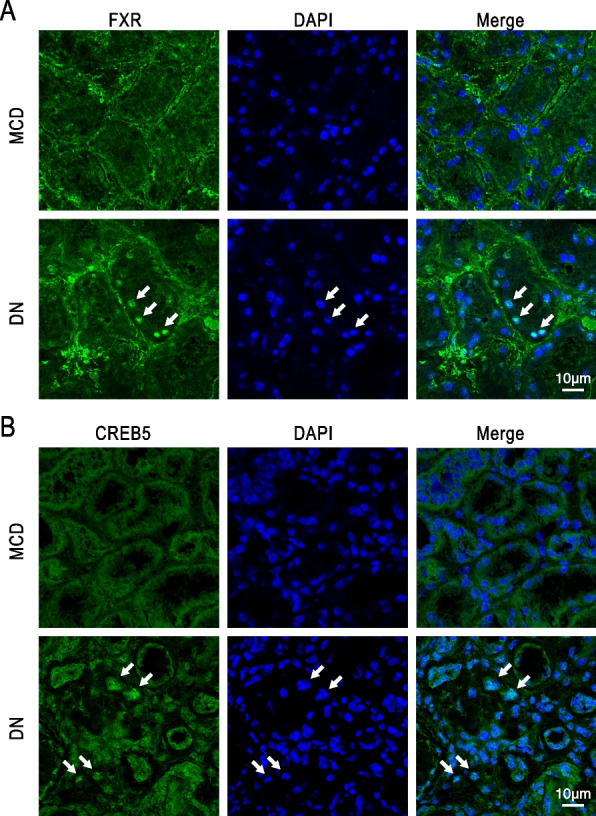
Representative images of FXR (**A**) and CREB5 (**B**) immunostaining (green) in the cortex region of the kidney from DKD and MCD patients. The white arrows denote the nuclear localization of FXR or CREB5

To validate the results of single nucleus RNA-seq and bioinformatic analysis in PCT cells, immunofluorescence staining was performed to examine the protein levels of FXR and CREB5 in kidney biopsies from 5 DKD patients and 6 MCD patients. MCD patients do not manifest overt tubular lesions and therefore serve as controls for the expression of FXR and CREB5. Compared with MCD samples, FXR staining intensity was markedly increased and exhibited nuclear accumulation in the PCT cells of the DKD samples (Fig. 4A). CREB5 staining was also increased and accumulated in the nuclei of PCT cells in the DKD samples (Fig. 4B).

### FXR and CREB5 protect PCT cells from injury in vitro

To investigate the functional role of increased FXR and CREB5 in PCT cell injury in diabetes, we performed a study in cultured HK2 cells, a cell line derived from human proximal tubular cells. We found that in the treatment with palmitic acid (PA) and advanced glycation end products (AGEs), which are commonly used in vitro models of DKD, the protein levels of FXR and CREB5 were significantly upregulated in the HK2 cells (Supplemental Fig. 4), suggesting that these two models are suitable for our purposes.

**Fig. 5:**
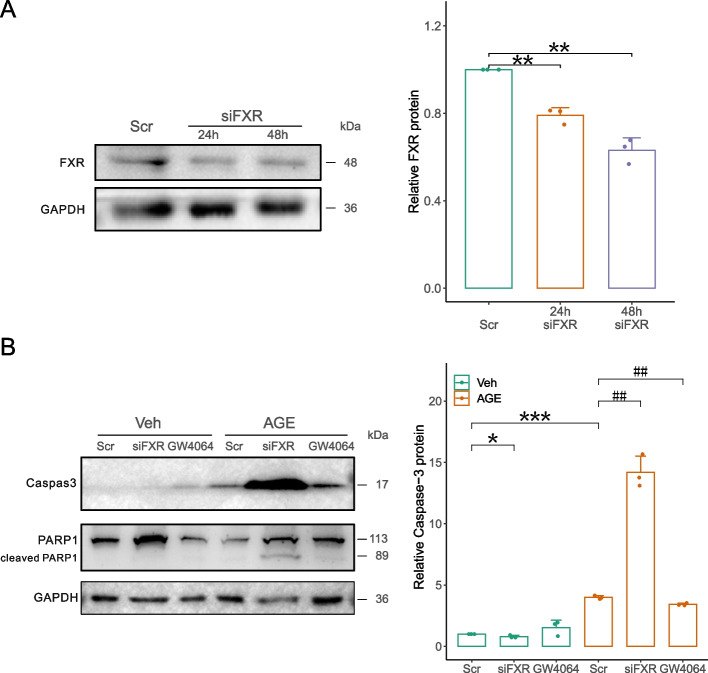
Knockdown of FXR sensitized HK2 cells to AGE-induced apoptosis. **A** Immunoblotting showed that siFXR effectively knocked down FXR in HK2 cells. **B** FXR knockdown aggravated AGE-induced activation of caspase-3 and PARP-1, as shown by an increase in their cleaved forms. All the results represent the data from three independent experiments. *p < 0.05; **p < 0.01; ^##^*p* < 0.01, statistical significance

**Fig. 6:**
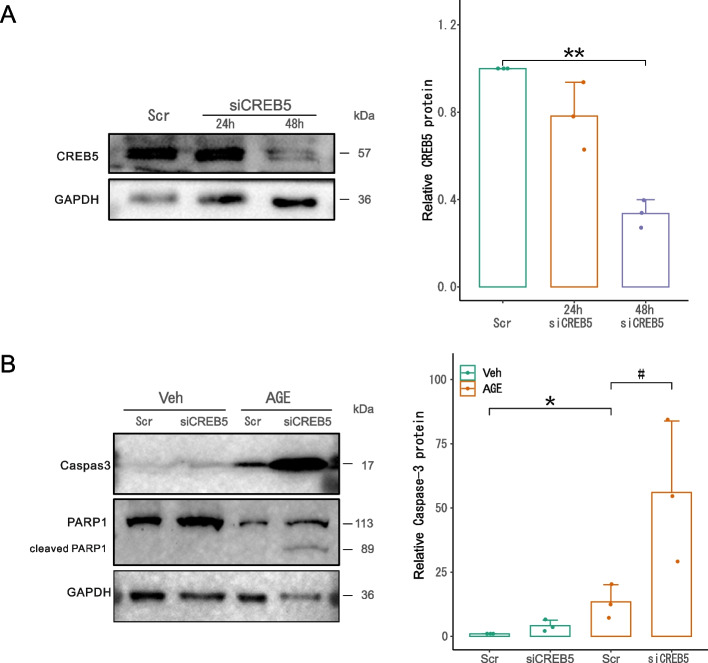
Knockdown of CREB5 sensitized HK2 cells to AGE-induced apoptosis. **A** Immunoblotting showed that siCREB5 significantly reduced CREB5 protein in HK2 cells. **B** CREB5 knockdown markedly enhanced the AGE-induced activation of caspase-3 and PARP1. All the results represent the data from three independent experiments. *p < 0.05; **p < 0.01; ^#^*p* < 0.05, statistical significance

The efficiency of siFXR (Fig. 5A) and siCREB5 (Fig. 6A) was tested and found to achieve ~ 80% and ~ 50% reduction of corresponding proteins in HK2 cells 48 h after transfection. In HK2 cells treated with AGEs, knockdown of FXR (Fig. 5B) or CREB5 (Fig. 6B) markedly increased AGE-induced caspase-3 cleavage. Consistently, the substrate of activated caspase-3, PARP1, was accordingly cleaved particularly in the AGE-treated cells that were pretreated with siFXR (Fig. 5B) or siCREB5 (Fig. 6B), suggesting that FXR and CREB5 protect HK2 cells against AGE-induced apoptosis. In Fig. 5B, we used FXR agonist GW4064 to test whether increasing FXR activity would further reduce AGE-induced caspase-3 activation (compared with Scr control), but we did not see this effect, suggesting that FXR is required but not sufficient to prevent AGEs-induced tubular cell apoptosis, at least, under the particular condition.

**Fig. 7:**
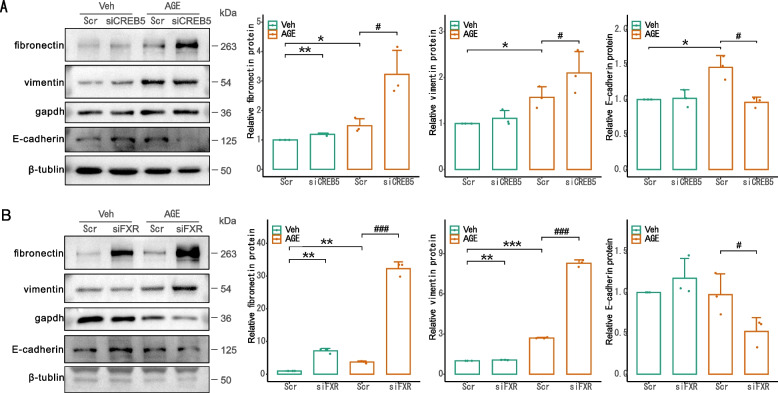
CREB5 and FXR knockdown aggravated AGE-induced EMT in HK2 cells. Immunoblotting of fibronectin, vimentin, E-cadherin in HK2 cells treated with siCREB5 (**A**) and siFXR (**B**) for 24 h, followed by treatment with 200 µg/ml AGE for 24 h. The results represent the data from three independent experiments. *p < 0.05; **p < 0.01; ***p < 0.001; #*p* < 0.05; ###*p* < 0.001. statistically significant

Since tubular cell injury is characterized by EMT, we wondered whether FXR and CREB5 are involved in EMT in HK2 cells. We performed immunoblotting to examine the change of EMT markers. As shown in Fig. 7, AGE treatment induced upregulation of fibronectin and vimentin while downregulation of E-cadherin in the HK2 cells (Fig. 7A, B), indicating that EMT was induced by AGE. In the cells treated with siFXR or siCREB5, the change of the fibronectin, vimentin, and E-cadherin became greater (Fig. 7A, B), demonstrating that FXR and CREB5 functioned to prevent EMT in the HK2 cells treated with AGE.

## Discussion

Previous beliefs held that DKD was primarily a glomerular disease, with kidney tubular injury being a secondary effect of the glomerular lesions. However, recent evidence suggests that proximal tubular injury is an early stage of DKD and may even play a role in promoting its progression [13]. Proximal tubular injury is characterized by hypoxia, mitochondrial dysfunction, abnormalities in fatty acid metabolism, impaired autophagy, inflammation, and EMT [14].

Through the construction of gene regulatory networks using single nucleus transcriptomes from kidney cortex samples of patients with early DKD and comparison of regulon activities between the DKD group and the control group, we discovered significant activation of FXR and CREB5 regulons in proximal tubule epithelial cells of diabetic patients.

FXR expression is mainly found in liver and intestine and is involved in the regulation of bile acid homeostasis, cholesterol, and carbohydrate metabolism [15-18]. In the kidney, FXR is expressed at moderate levels in proximal tubular epithelial cells. In kidney diseases such as DKD, FXR has been shown to protect tubular cells through its anti-inflammatory, antifibrotic, antilipogenic, and antioxidant effects [19-22].

CREB proteins belong to a large gene family that consists of 6 subfamilies (CREB, B-ATF, ATF2, ATF3, ATF4 and ATF6) [23]. The CREB/CREB1 subfamily has been extensively studied. It is widely expressed and plays a crucial role in numerous important cellular signaling pathways, including cellular metabolism, cell cycle progression, survival, and responses to extracellular stimuli [24-28]. CREB5, a member of the ATF2 subfamily, has not been studied in the kidney except for one report indicating its potential involvement in mediating fibronectin deposition in kidney fibrosis [29].

The activation of FXR and CREB5 in kidney PCT cells from DKD patients was further validated with immunofluorescence staining. This study is the first to show the specific activation of CREB5 in proximal tubular cells in early DKD. In cultured HK2 cells, we confirmed the protective role of FXR by demonstrating that FXR knockdown aggravated AGE-induced apoptosis. We also suggest that the activation of CREB5 in response to diabetic conditions serves as a protective mechanism in proximal tubular cells, with an antiapoptotic effect, similar to CREB1.

We also investigated the effect of FXR and CREB5 on EMT in HK2 cells treated with AGEs. EMT has been observed in tubular epithelial cells in various kidney diseases and is thought to contribute to kidney fibrosis through the generation of fibroblasts from tubular cells [30]. EMT is a survival strategy employed by cancer cells to avoid cell death, including apoptosis [31]. TGF-beta-induced apoptosis and EMT in tubular cells are two distinct pathways [32]. We found that FXR and CREB5 ameliorated EMT in HK2 cells treated with AGEs, indicating that the anti-EMT effect is an additional mechanism underlying the protective effect of FXR and CREB5 on tubular epithelial cells in DKD.

Hyperglycemia is the primary cause of DN, but it alone is not sufficient to cause DN because more than half of diabetic patients do not develop diabetic nephropathy and some others whose blood glucose is well controlled still have worsening DN, demonstrating that some factors that may be associated with genetic susceptibility to DN are also required for DN development. In the early stage of DN, AGEs and fatty acids, which are known to be harmful to tubular and other cells in the kidney, are already elevated as the consequence of hyperglycemia. In the present study, we observed that high glucose is not able to induce FXR and CREB5 in HK2 cells (Supplemental Fig. 5) in contrast with AGEs and PA, suggesting that hyperglycemia, AGEs, and fatty acids have distinct impacts on kidney cells in early DN. Therefore, the role of hyperglycemia in the early stage of DN includes not only the direct damage on kidney cells but also the induction of AGEs, fatty acids, RAS, hemodynamic change, etc., all of which act concertedly to promote DN development.

In conclusion, our study identified several cell type-specific regulons in kidney tissue from patients with early-stage DKD. Some of these regulons, such as FXR and CREB5 in PCT cells, may offer potential targets for early intervention in DKD.

## Supplementary Information

**Additional file 1.**

## Data Availability

The single nucleus RNA-seq data can be accessed in the GEO database with the provided accession number, GSE131882.
