# Supplementary material for: Regulon analysis identifies protective FXR and CREB5 in proximal tubules in early diabetic kidney disease
Source: BMC Nephrol. 2023 Jun 19;24:180. doi: 10.1186/s12882-023-03239-6 (PMC10278299; doi:10.1186/s12882-023-03239-6)
Supplement: Supplementary file 1 — Additional file 1. [file 12882_2023_3239_MOESM1_ESM.pdf]

Regulon analysis identifies protective FXR and CREB5 in proximal tubules in early diabetic kidney disease

Wanting Shi[1], Weibo Le[1*], Qiaoli Tang[1,2], Shaolin Shi[1#], Jingsong Shi[1#]*

[1]National Clinical Research Center for Kidney Disease, Affiliated Jinling Hospital, Medical School, Nanjing University, Nanjing, China

[2]Department of Nephrology, the First Affiliated Hospital of University of Science and Technology of China, Hefei, China

[*] These authors contributed equally.

[#] Correspondence:

Jingsong Shi, PhD

~~Research institute of Nephrology~~

305 East Zhongshan Road, Nanjing 210002

China

Email: shijs1982@hotmail.com

Shaolin Shi, PhD

~~Research institute of Nephrology~~

305 East Zhongshan Road, Nanjing 210002

China

Email: shaolin.shi@nju.edu.cn

Supplemental table 1: siRNA sequences

siRNA	Sense	Anti-Sense
siFXR	GAUUGUUACUUCAAUUCUA	UAGAAUUGAAGUAACAAUCTT
siCREB5	AACAGTATTCTGTAGGATCTA	TAGATCCTACAGAATACTGTT

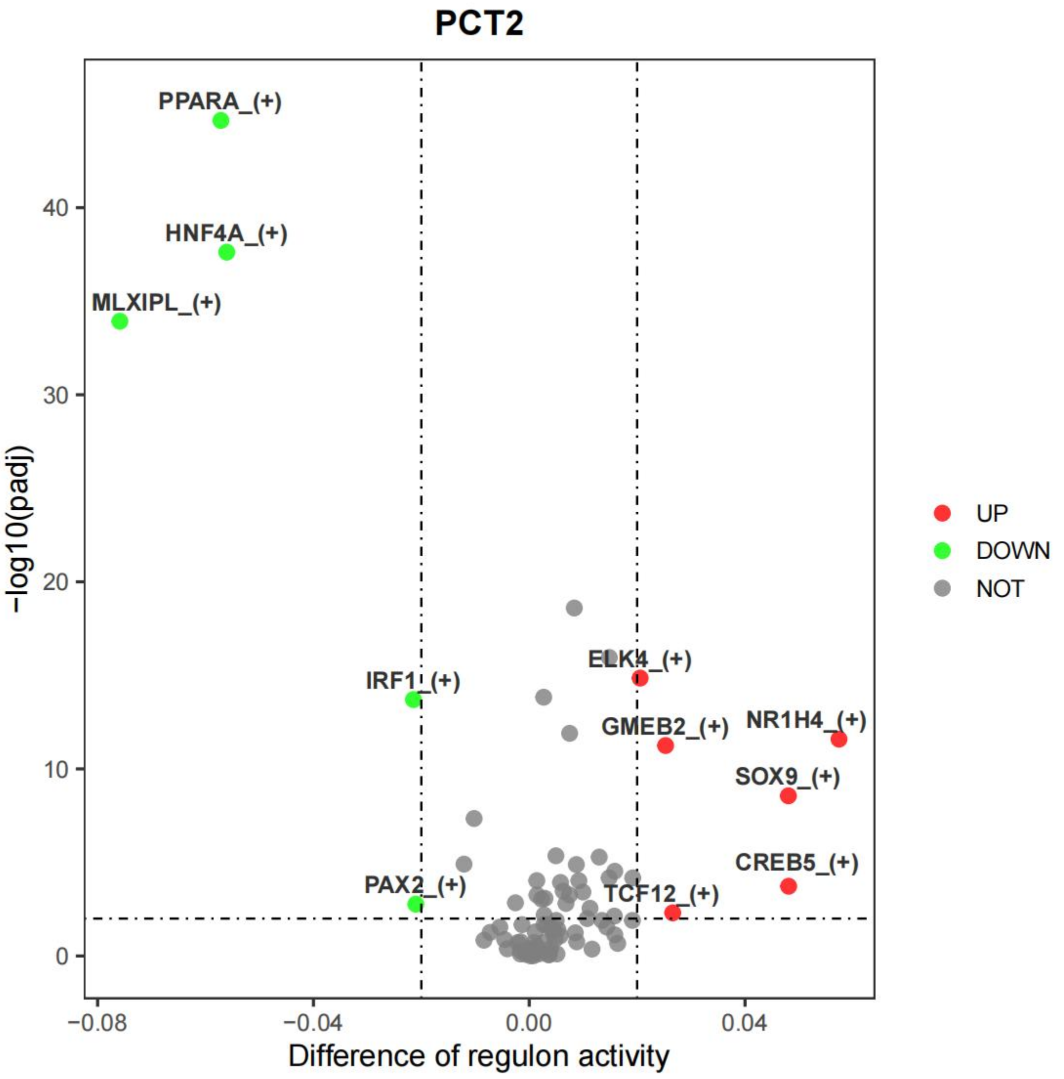

Supplemental figure 1: The regulon activity changes in kidney convoluted proximal tubule epithelial cell type 2 in diabetic patients; PCT2, proximal convoluted tubule epithelial cell type 2.

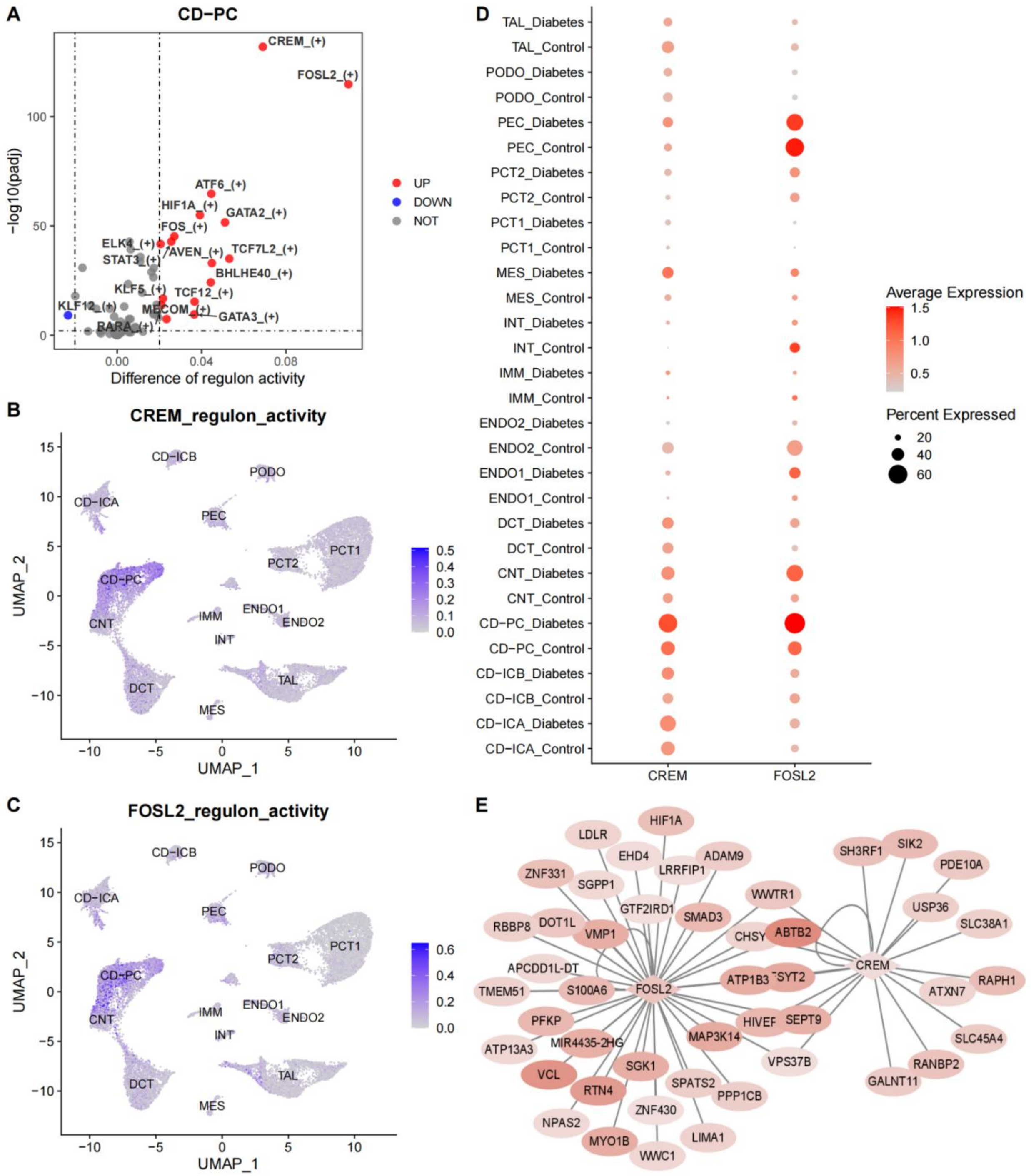

Supplemental figure 2: (A) The regulon activity changes of the kidney collecting duct principal cells in the diabetic patients; CD-PC, collecting duct principal cells; (B) The regulon activities of CREM across kidney cell types; (C) The regulon activities of FOSL2 across kidney cell types; (D) mRNA expression of CREM and FOSL2 across kidney cell types in diabetic and control conditions; (E) Gene regulatory network of TF CREM and FOSL2 and their target genes. The pink color indicates that the mRNA of gene was upregulated in diabetic principal cells, and the intensity of color denotes relative upregulation across the genes.

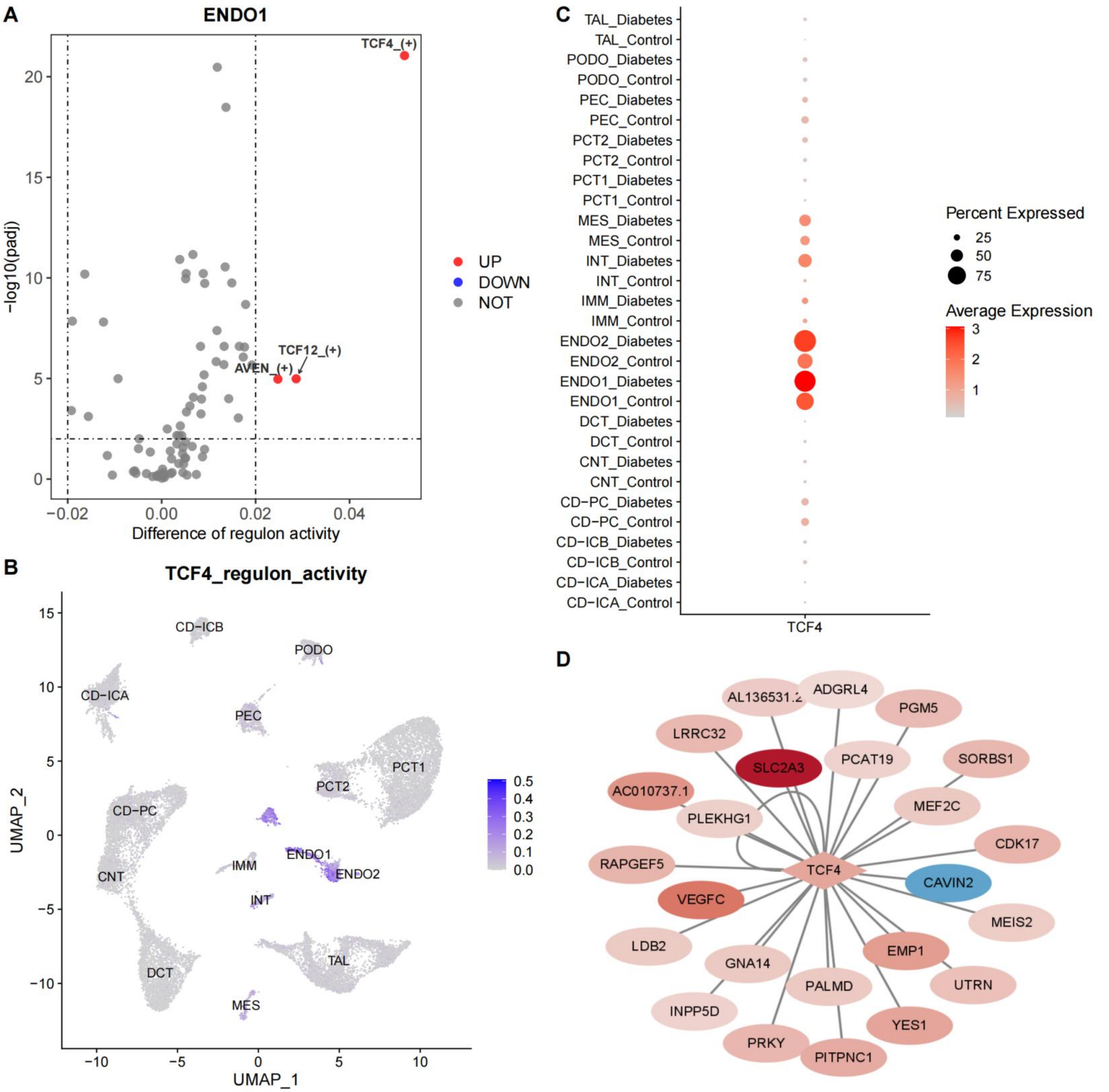

Supplemental figure 3: (A) The regulon activity changes in the glomerular endothelial cells. (B) The TF activities of TCF4 across kidney cell types; (C) The mRNA expression of TCF4 was also upregulated in the diabetic glomerular endothelial cells; (D) Gene regulatory network of TF TCF4 and its target genes. The pink color denotes upregulation while blue downregulation of the gene. The intensity of color denotes relative change across the genes.

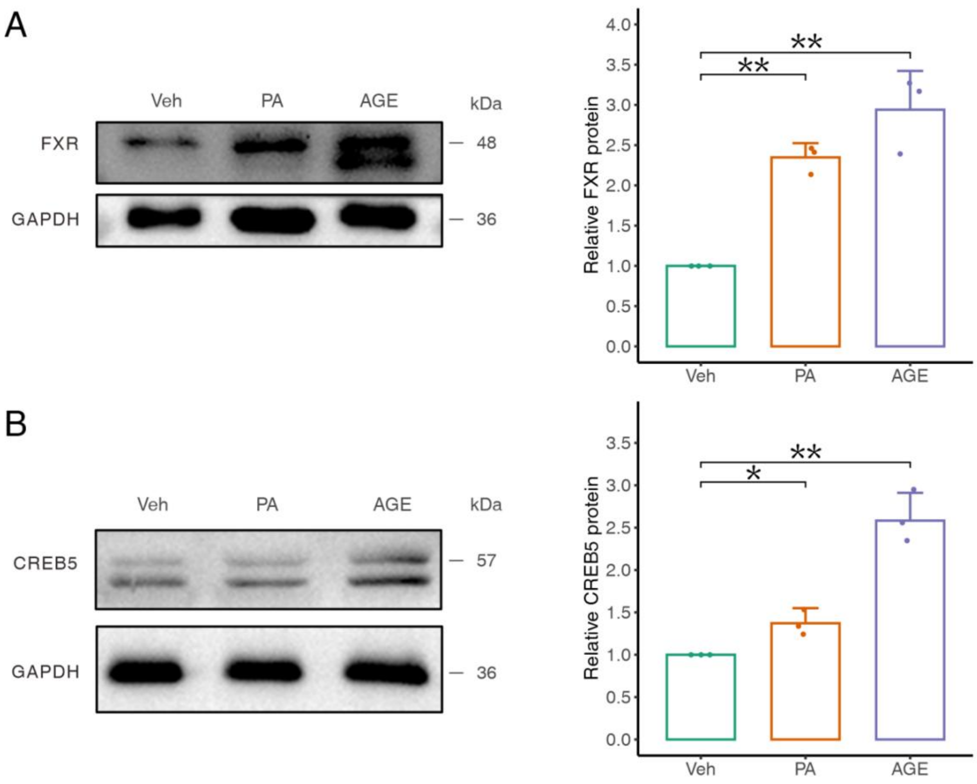

*Supplemental figure 4: In vitro models of palmitic acid (PA) and advanced glycation end products (AGEs) with HK2 cells. A. Immunoblotting showed that PA and AGEs both increased the protein level of FXR. B. AGEs upregulated CREB5 protein expression in HK2 cells. The results represented the data from three independent experiments. * p<0.05; ** p<0.01: statistical significance.*

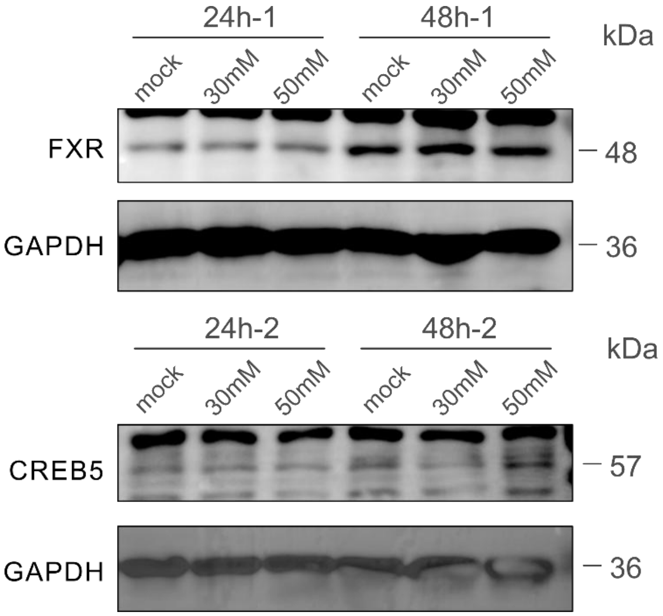

Supplemental figure 5: High glucose did not induce upregulation of FXR and CREB5 in HK2 cells.

Figure 5A

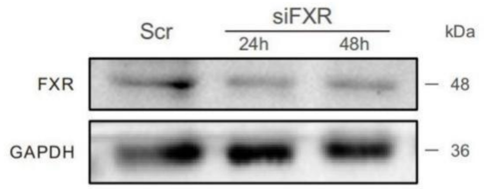

Original blots

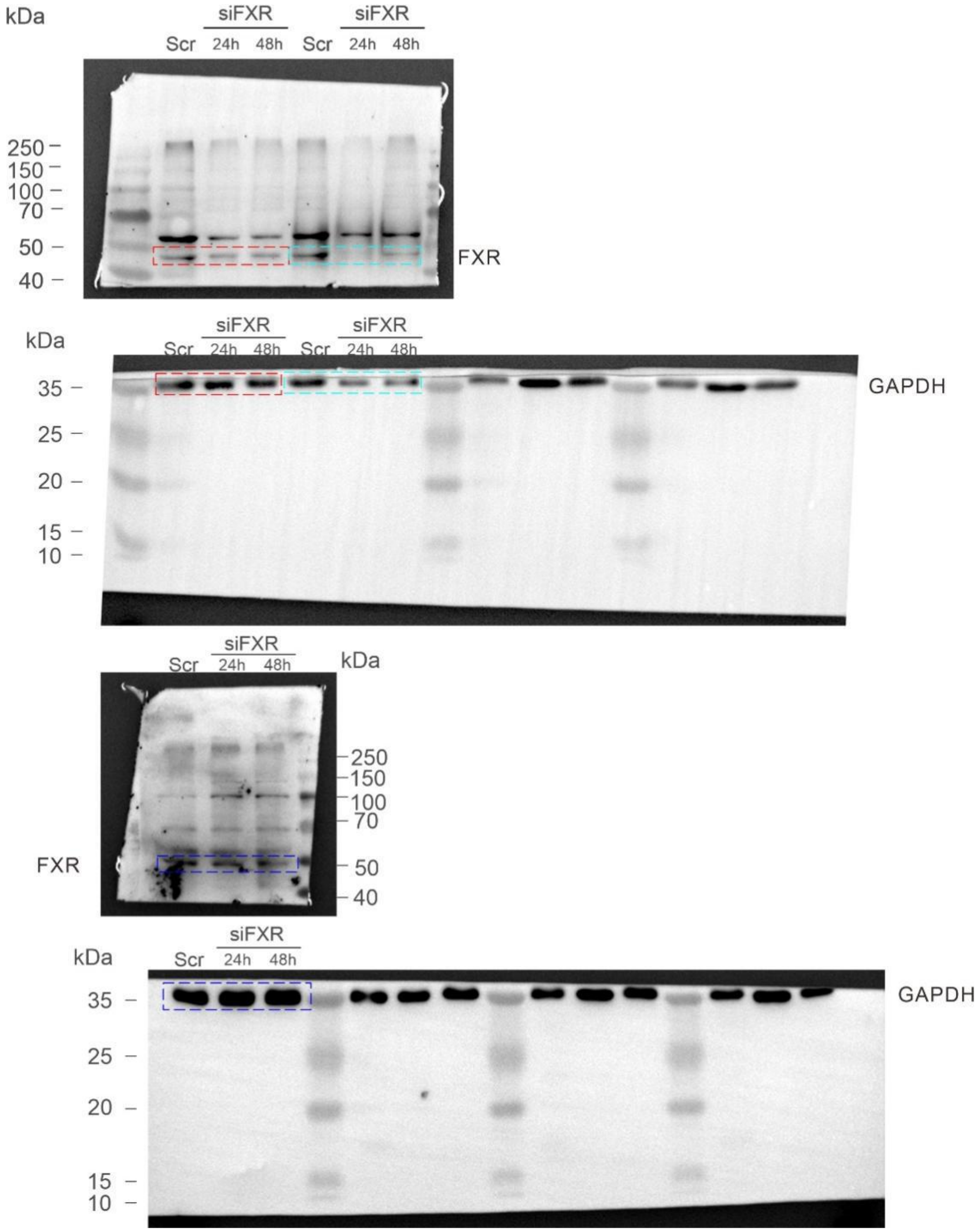

Supplemental Figure 6: Uncropped, full-length images of Western blot membranes from Figure 5A. The red dotted boxes highlight the blots displayed in Figure 5A, while the turquoise and blue dotted boxes represent the second and third replicates, respectively. Blots were cut into two or three pieces and hybridized with different antibodies for proteins with a known size for more efficient use of the blots.

Figure 5B

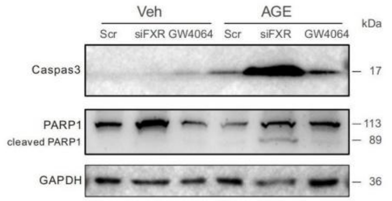

Original blots

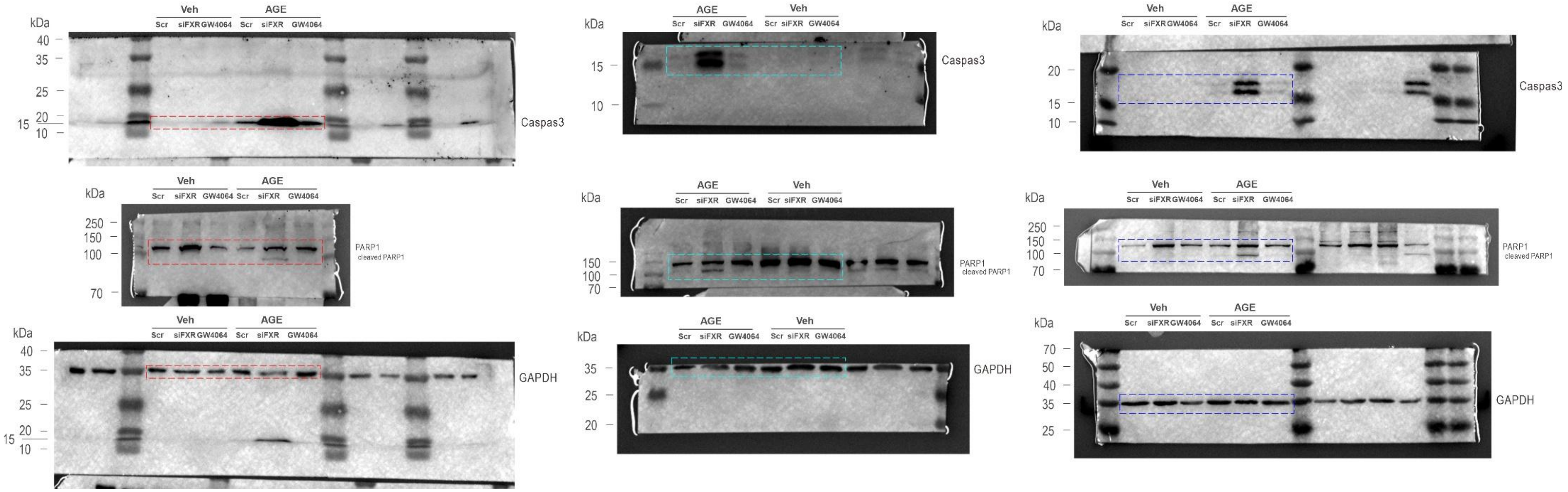

Supplemental Figure7: Uncropped, full-length images of Western blot membranes from Figure 5B. The red dotted boxes highlight the blots displayed in Figure 5B, while the turquoise and blue dotted boxes represent the second and third replicates, respectively. Blots were cut into two or three pieces and hybridized with different antibodies for proteins with a known size for more efficient use of the blots.

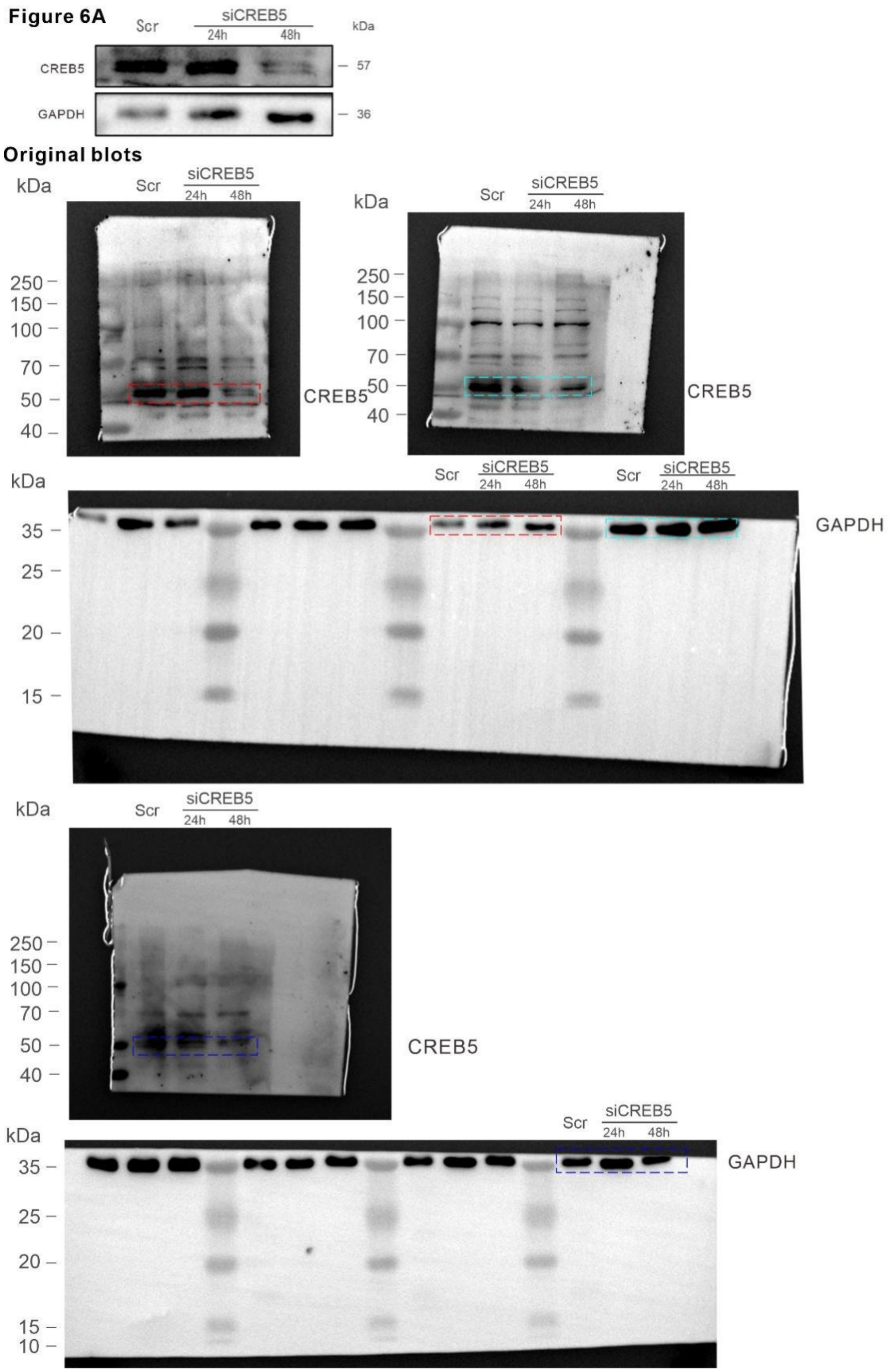

Supplemental Figure 8: Uncropped, full-length images of Western blot membranes from Figure 6A. The red dotted boxes highlight the blots displayed in Figure 6A, while the turquoise and blue dotted boxes represent the second and third replicates, respectively. Blots were cut into two or three pieces and hybridized with different antibodies for proteins with a known size for more efficient use of the blots.

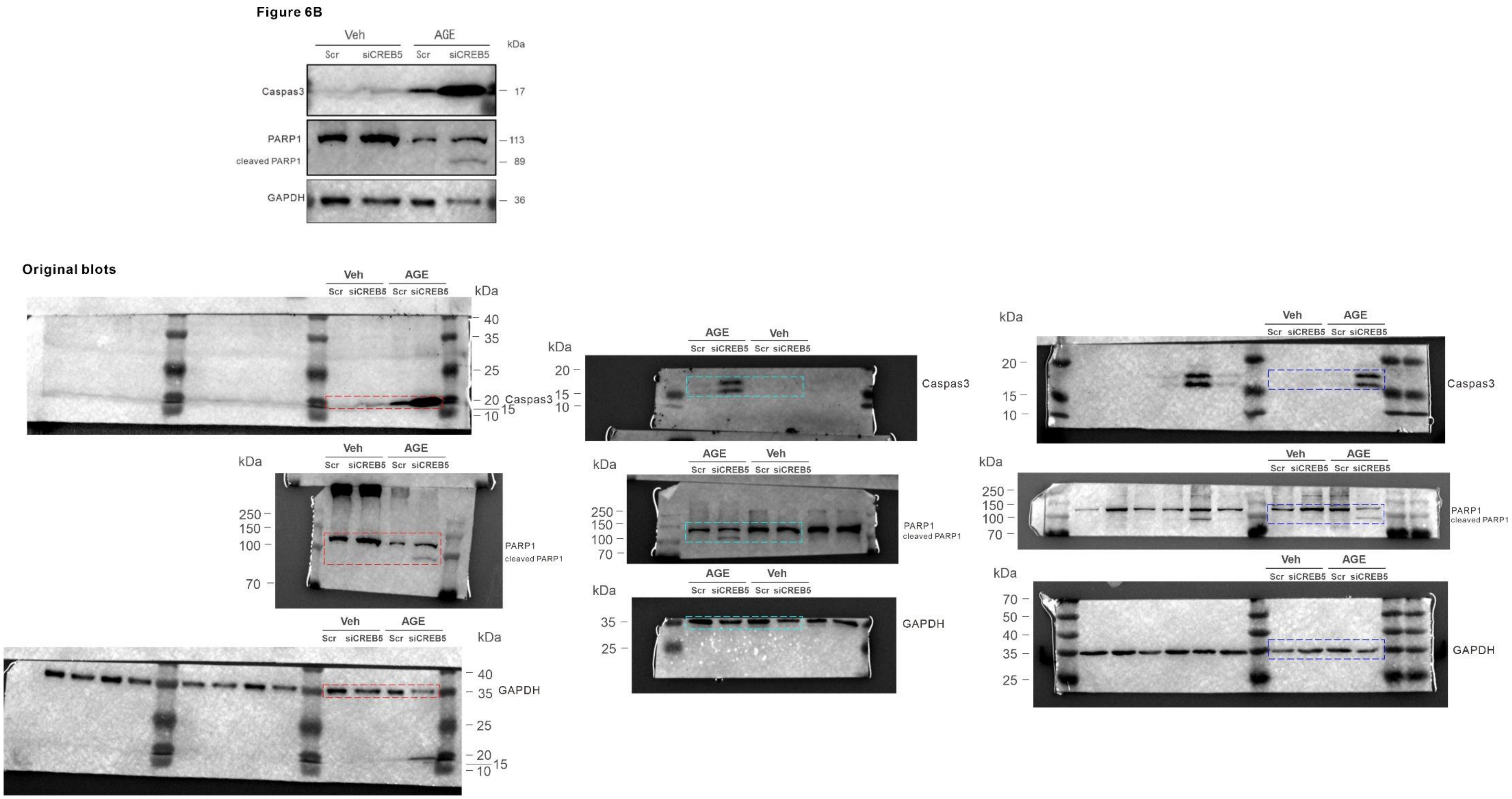

Supplemental Figure 9: Uncropped, full-length images of Western blot membranes from Figure 6B. The red dotted boxes highlight the blots displayed in Figure 6B, while the turquoise and blue dotted boxes represent the second and third replicates, respectively. Blots were cut into two or three pieces and hybridized with different antibodies for proteins with a known size for more efficient use of the blots.

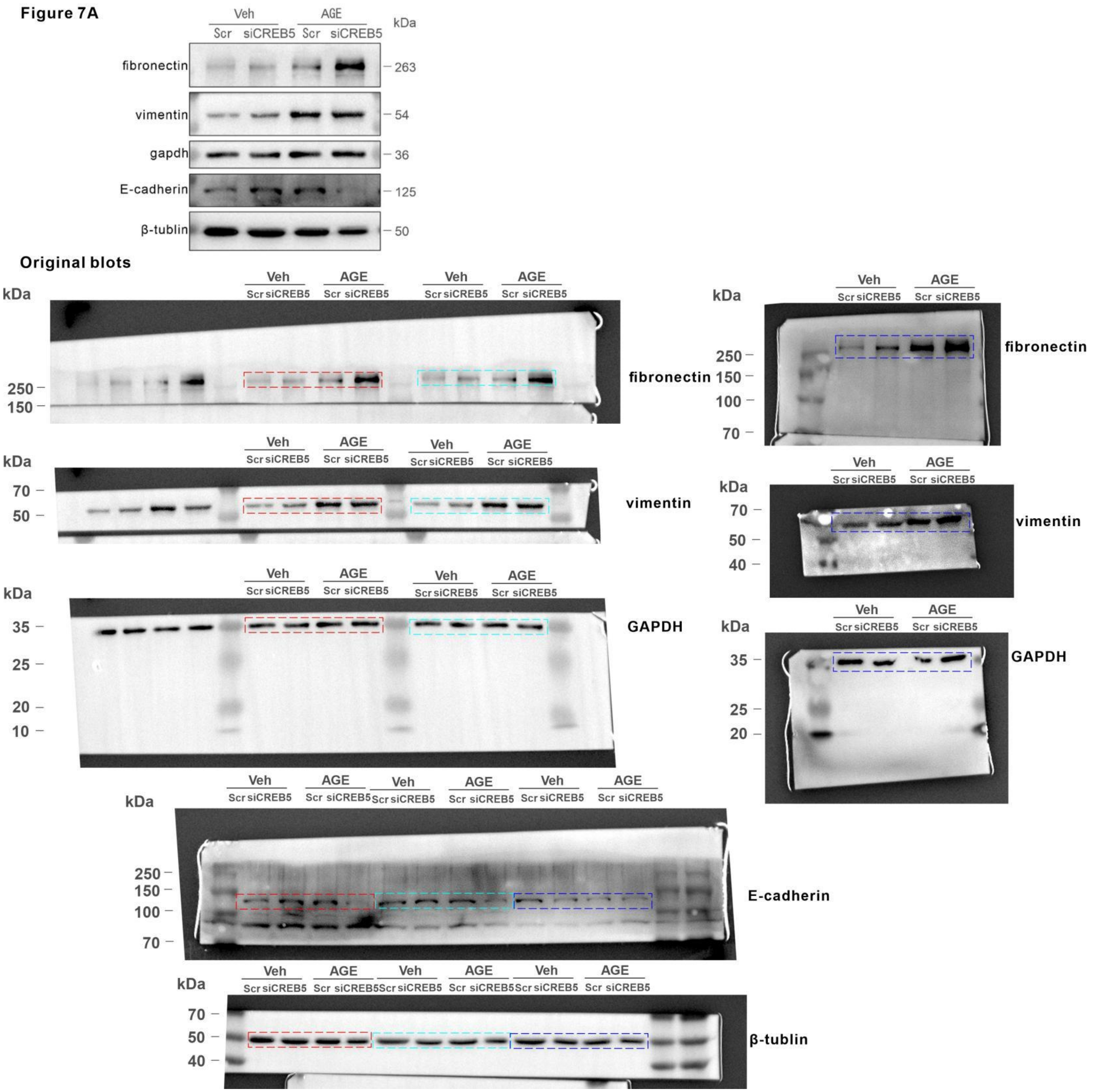

Supplemental Figure 10: Uncropped, full-length images of Western blot membranes from Figure 7A. The red dotted boxes highlight the blots displayed in Figure 7A, while the turquoise and blue dotted boxes represent the second and third replicates, respectively. Blots were cut into two or three pieces and hybridized with different antibodies for proteins with a known size for more efficient use of the blots.

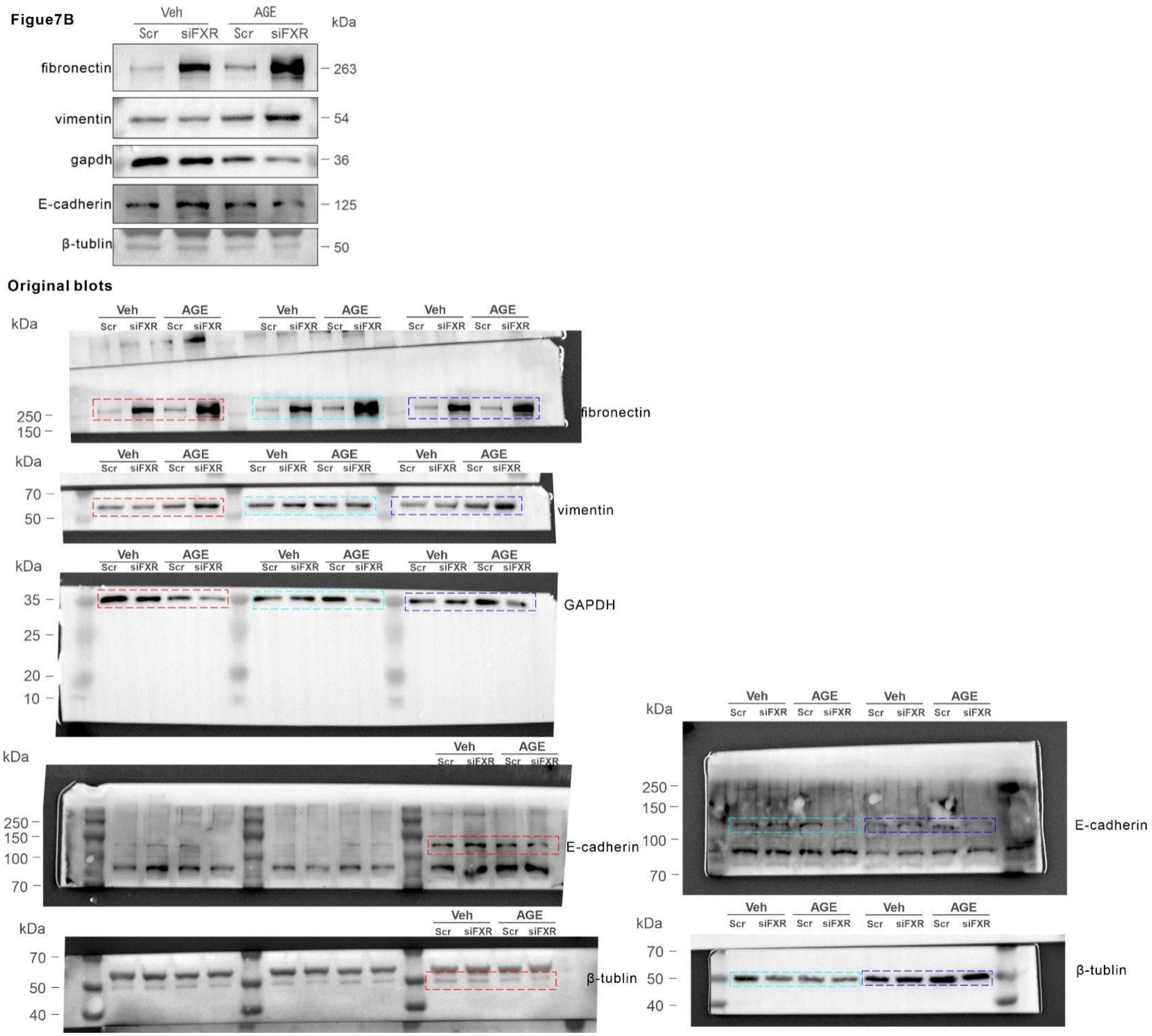

Supplemental Figure 11: Uncropped, full-length images of Western blot membranes from Figure 7B. The red dotted boxes highlight the blots displayed in Figure 7B, while the turquoise and blue dotted boxes represent the second and third replicates, respectively. Blots were cut into two or three pieces and hybridized with different antibodies for proteins with a known size for more efficient use of the blots.

Supplemental Figure 4A

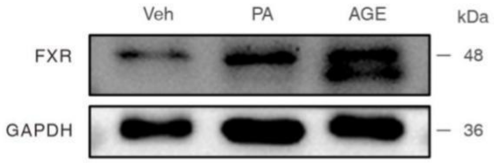

Original blots

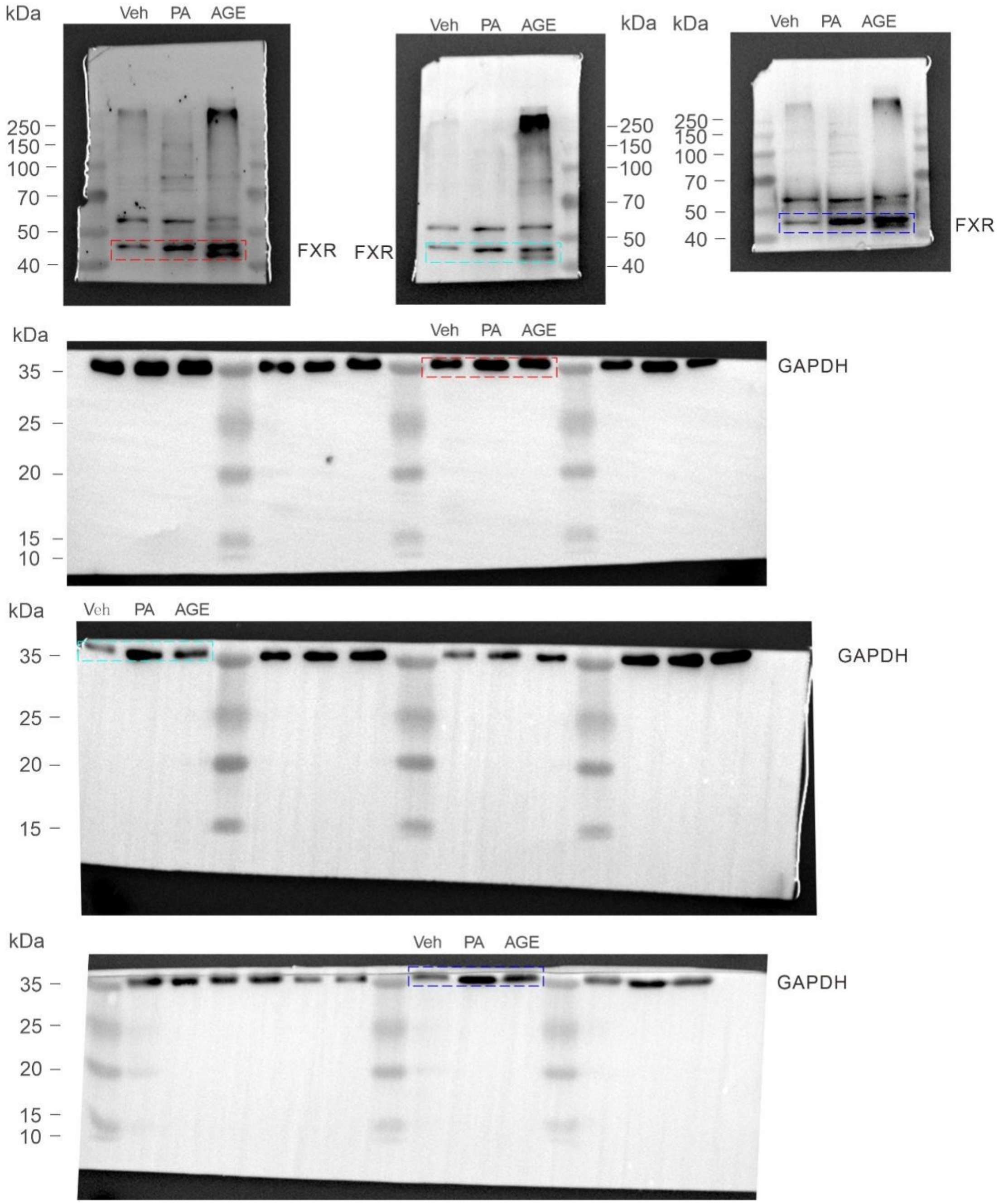

Supplemental Figure 12: Uncropped, full-length images of Western blot membranes from Supplemental Figure 4A. The red dotted boxes highlight the blots displayed in Supplementary Figure 4A, while the turquoise and blue dotted boxes represent the second and third replicates, respectively. Blots were cut into two or three pieces and hybridized with different antibodies for proteins with a known size for more efficient use of the blots.

Supplemental Figure 4B

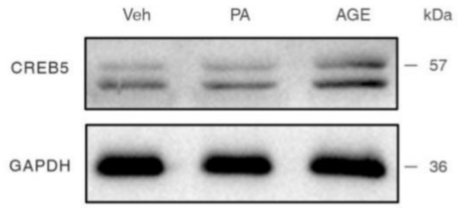

Original blots

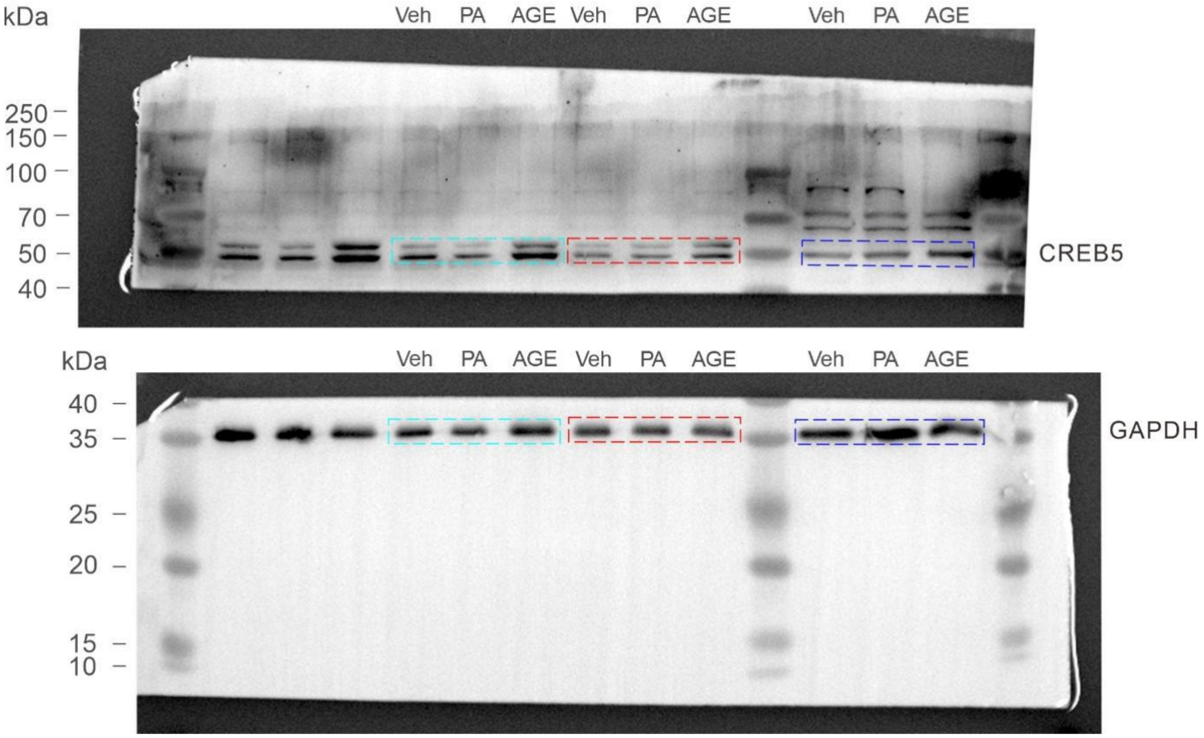

Supplemental Figure 13: Uncropped, full-length images of Western blot membranes from Supplemental Figure 4B. The red dotted boxes highlight the blots displayed in Supplementary Figure 4B, while the turquoise and blue dotted boxes represent the second and third replicates, respectively. Blots were cut into two or three pieces and hybridized with different antibodies for proteins with a known size for more efficient use of the blots.

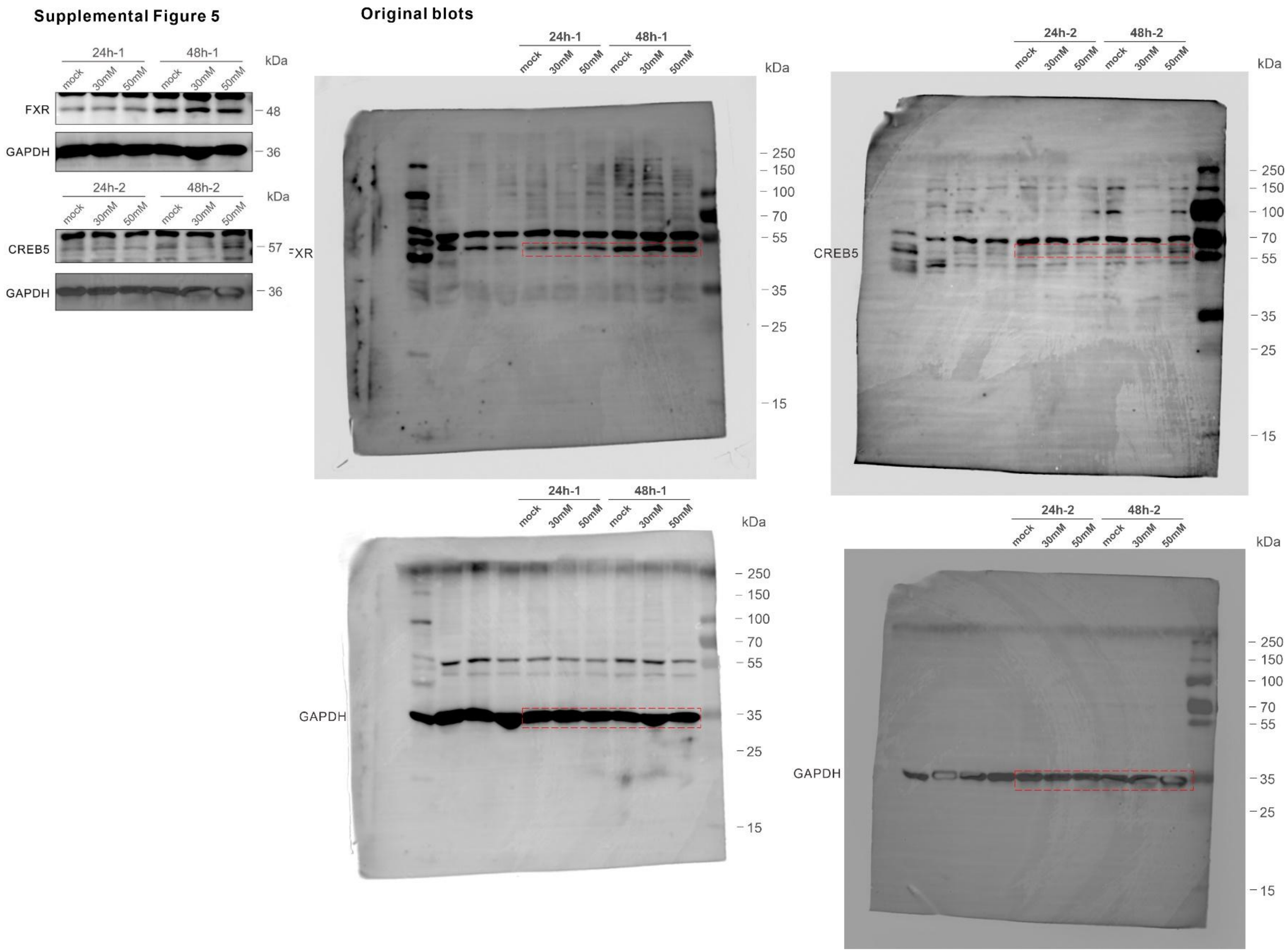

Supplemental Figure 14: Uncropped, full-length images of Western blot membranes from Supplementary Figure 5. The red dotted boxes highlight the blots displayed in Supplemental Figure 5.
